# A Threat-and-Defense Perspective on the Psychological Dynamics Behind the Covid-19 Pandemic

**DOI:** 10.5334/irsp.792

**Published:** 2023-12-28

**Authors:** Chiara Annika Jutzi, Robin Willardt, Johannes Klackl, Janine Stollberg, Stefan Reiss, Eva Jonas

**Affiliations:** 1 University of Salzburg, Austria; 2 ETH Zürich, Switzerland

**Keywords:** Threat and Defense, pandemic, Covid-19, existential questions

## Abstract

In this review, we analyse the Covid-19 pandemic in the light of the General Process Model of Threat and Defense (GPMTD; Jonas et al., 2014) and describe motivational and affective consequences resulting from the psychological threat elicited by the pandemic: Covid-19 did not only abruptly change everyday life, but also confronted people with existential questions. This led to the experience of discrepancies that people could not resolve, triggering an aversive state of anxious inhibition. People were motivated to overcome this anxiety using defensive behaviours that re-establish approach motivation. Such defensive behaviours include conspiratorial thinking and increased ingroup support. While describing the pandemic, we review evidence in favour and against the model to develop concrete suggestions to effectively promote non-destructive reactions to manage affective-motivational challenges resulting from the pandemic. In a final outlook, we conclude that the evidence generated may be used to analyse and react to future crises and their related psychological threats.

Since, in January 2020, the World Health Organization declared Covid-19 “a global public health emergency” with the “highest level of alarm” (World Health Organization, 2020a), millions of people have died from the virus or still suffer from its long-term effects. Vaccination programmes have been rolled out across the globe with a worldwide initiative (COVAX) to distribute vaccines and other medical resources to economically challenged countries (World Health Organization, 2020b). Next to the immediate health threat posed by the virus, societal measures to counteract the pandemic have created threats in the form of social isolation, economic insecurity, and uncertainty regarding the future. Thus, the pandemic created difficult circumstances and an unexpected new reality that could not be resolved by adjusting individual behaviour. More than that, the pandemic increased the salience of questions related to life and death, freedom and responsibility, relationships with others, and meaning (Bland, 2020; Koole et al., 2006). These questions are the “most profound and universal existential conflicts people must face” (Koole et al., 2006, p. 213). The pandemic relentlessly exposed people to these unanswerable questions and thus violated fundamental human needs. This violation in turn led to the experience of existential threat (Jonas et al., 2014). Using it as a case study, we describe the typical human threat response to existential threats within the framework of our General Process Model of Threat and Defense (GPMTD). Precisely, we argue that the pandemic activated existential threats and use the GPMTD to describe and analyse the pandemic’s affective, motivational, and behavioural consequences. We conclude with an outlook that views other global crises through the lens of the GPMTD.

## Overview

The current article explores people’s reactions to the pandemic using the GPMTD (Jonas et al., 2014), which offers a synoptic account of decades of social psychological threat and defense research. The first of four main sections outlines how the GPMTD explains common responses to the pandemic. We then illustrate that the pandemic highlighted different kinds of existential threats. Next, we elaborate on our recent cognitive, neural, and affective-motivational research findings. Lastly, we discuss how insights from our research may aid a broader understanding of how humanity can better cope with global crises such as the pandemic, climate change, and other threats.

### The General Process Model of Threat and Defense

#### Threats as Discrepancies

The pandemic dramatically changed daily life worldwide in early 2020. At the same time, the measures taken to combat Covid-19 increased peoples’ feelings of threat. The GPMTD indicates that these feelings ultimately result from a “discrepancy between an expectation or desire and current circumstances” (Jonas et al., 2014, p. 229). While the GPMTD integrates different theories into one account, this integration is bound to a broad definition of threat as a discrepancy.^1^

Additionally, the GPMTD further distinguishes two types of discrepancy or mismatches: epistemic (a divergence from the expected) and motivational (a violation of a need). In this frame, the pandemic was neither expected nor desired, leading to discrepancies regarding basic human needs for autonomy, social belonging, competence, predictability, control, and acceptance (Deci & Ryan, 2012; Dweck, 2017). When these basic needs are frustrated and cannot be resolved, people have to “come to terms with these basic facts of life” (Koole et al., 2006; p. 212). In our model, we interpret such threats as existential. Perhaps the most prominent existential threat, death through virus infection, has been joined by the side effects of the pandemic lockdown measures, such as disruption of the world economy. Social distancing has limited our means of contact and inclusion and made us aware of existential loneliness. Economic uncertainties and the impossibility of certain work getting done have led many to worry about losing their job. Furthermore, containment strategies have involved restrictions on personal freedoms and burdened healthcare capacities, which have increased different kinds of motivational discrepancies (Reiss et al., 2020). The existential nature of the pandemic still portrayed a psychological threat to all members of society – even though disadvantaged population groups have been affected more by many factors such as cramped living conditions and reduced access to resources (Sommet & Elliot, 2023).

#### Threat Activates the Behavioural Inhibition System

The GPMTD assumes that the threat-response process is based on the (de)activation of the two basic human motivation systems proposed by Reinforcement Sensitivity theory (Gray & McNaughton, 2000): the behavioural inhibition system (BIS) and the behavioural approach system (BAS). Expectancy and need discrepancies activate the BIS, which results in heightened anxiety, arousal, attentional vigilance, and avoidance (Corr et al., 2013). The system monitors perceptual inputs and detects discrepancies between target status and actual reality. This target status is determined by the desires for control, life certainty and meaning. Upon detecting such a discrepancy, goal pursuit is inhibited, allowing people to acquire more information via heightened attention (Gray & McNaughton, 2000). BIS avoidance is better described as a passive rather than an active state as it does not motivate an active approach or avoidance behaviour (e.g., flight). This passive nature distinguishes the BIS from the fight-flight-freeze system (FFFS), which mediates active avoidance behaviour, and the BAS, which organises reward-oriented approach behaviour (Gray & McNaughton, 2000).

The pandemic provides no option to escape the elicited threat state actively. Need violations are omnipresent and multifaceted and likely to trigger the BIS. The BIS-evoked avoidance and vigilance processes often manifest in parallel, as they do with news reports on the Covid-19 pandemic. For example, people often manage uncertainty by avoiding unstable phenomena (Rutjens et al., 2013). The simultaneous presence of vigilance and avoidance has become apparent during the Covid-19 pandemic. For example, the news was entirely dominated by Covid-19, and at the same time, people wanted to avoid the distressing news (Siebenhaar et al., 2020).

#### Defensive Responses Activate the Behavioural Activation System

People typically experience BIS activation following threat as immediate discomfort and are motivated to overcome this aversive state by engaging in different responses that reflect activation of the BAS. According to the GPMTD, the BAS allows people to turn to rewarding and attractive matters and away from anxiety (Gray & McNaughton, 2000), producing an affective-motivational state of positive determination and activation.

Defensive responses may be categorised as actively tackling a problem at hand (*direct resolution*) or indirectly addressing underlying needs (*palliative responses*). Direct resolution strategies involve people’s engagement in problem-solving (potentially fostering lasting solutions; Park & Millora, 2012) and restoration of the threatened need. Palliative responses distract people from the threat cognitively by engaging in a threat-unrelated domain to relieve anxiety without solving the threat (Jonas et al., 2014; Quirin et al., 2019). This distinction is rooted in earlier accounts of emotion regulation.^2^

##### Resolution and Palliation as Defensive Responses

Much human behaviour aims to solve problems, which can reduce the anxiety the problems cause and re-establish goal pursuit. However, when a threat cannot be resolved, as in the case of existential threats, problem-solving in a direct sense is not possible. People, therefore, often turn away from the source of the threat and towards more edifying things that restore approach motivation. Research suggests that this kind of palliation can be achieved by escaping into abstract defenses (Jonas et al., 2014) such as affirming ingroups (Fritsche et al., 2008), worldviews (Burke et al., 2010), values, and ideals (Kay & Eibach, 2013). To directly resolve discrepancies stemming from the Covid-19 pandemic, people could adhere to hygienic and social distancing rules and get vaccinated. Palliation, in contrast, may be achieved by defending one’s ingroup by exhibiting solidarity with other group members. On the other hand, palliative defenses can also be associated with more negative consequences, such as spreading conspiracy theories (van Prooijen & Douglas, 2017). While one could observe many acts of solidarity throughout the pandemic, Covid-related conspiracy theories have been used by some to reduce feelings of uncertainty (van Mulukom et al., 2020; Lee & Koo, 2022) and provide alternative explanations and structure in an uncertain time (van Prooijen & Acker, 2015).

### The Pandemic as a Global Threat

#### Covid-19 Threatens Existential Needs

The pandemic threatens many existential needs related to continued existence, control, certainty, meaning, belonging, and freedom. The GPMTD suggests that psychological need violations produce a threat-and-defense cascade. We propose that in case of the pandemic, in a similar vein, multiple distinct existential threats were made salient by the pandemic and thus different stages of threat processing were activated.

##### Mortality Salience

The pandemic has reminded us of our mortality, as being infected could and still can have serious consequences, including death. While this is true for many diseases, Covid-19 is ubiquitous and has received constant media attention for a long time, creating high and steady salience of a specific cause of death in people’s minds.

Terror management theory says that this exposure can proximally lead to the suppression of death-related thoughts. While some may have played down the virus’ danger by spreading misinformation, others may have distracted themselves through alcohol consumption, eating, or television viewing (Pyszczynski et al., 2020). However, the widespread use of social distancing to contain the virus hampered the pursuit of close interpersonal relationships. For many, self-esteem was undermined by the loss of their source of income or the inability to pursue meaningful activities such as hobbies. The diminished access to, or loss of, these much-needed anxiety buffers may help explain the rising reports of anxiety, depression, and stress throughout the pandemic (Pyszczynski et al., 2020).

##### Freedom and Personal Control

The pandemic has deeply shaken many people’s feelings of control over important aspects of life. Such a lack of control can stem from sudden personal freedom restrictions. While these restrictions to freedom elicited reactance and approach motivation (Mühlberger et al., 2017), personal helplessness and uncontrollability are strongly related to depression, anxiety, and long-term health impairment (Rodin & Langer, 1977). During the pandemic containment measures have restricted people’s freedom, which may have been a source of reactance (Reiss et al., 2020).

An existential personal control threat can motivate people to restore a sense of control on the social level (i.e., by acting as group members; Fritsche et al., 2008; Stollberg et al., 2017). Thinking and acting as ingroup members restores collective agency by feeling that “as a group, *we* can” act successfully through joint effort when *I* cannot do this alone (Fritsche et al., 2013; Stollberg et al., 2015). In addition, people can maintain a general sense of things being under control through a motivated perception of structure in the world (Kay et al., 2008), benevolent governments (Kay et al., 2008), well-structured scientific theories (Rutjens et al., 2013), and conspiracies (Douglas et al., 2017).

##### Uncertainty

“A striking feature of the Covid-19 pandemic is its unprecedented nature. This ushers in a profound uncertainty” (Kruglanski et al., 2021, p. 285). Three years into the pandemic, it remains hard to predict how infection rates will develop or whether new lockdown measures will have to be put in place in the future. Uncertainty includes the unpredictability of the future and personal concerns about current roles and identity in general (Hogg, 20021a). Uncertainty also increases identification with groups and raises support for “radical group action” (Hogg & Adelman, 2013, p. 440; Hogg, 2021b), which are plausible catalysts of approach motivation (McGregor & Jordan, 2007).

##### Meaning

Covid-19 has thoroughly shaken our collective understanding of the world as acquired knowledge about life has become non-adaptive in handling the pandemic. Humans create meaning by assigning relationships between people, places, objects, and ideas (Proulx & Inzlicht, 2012). Experiences that violate expected relationships elicit an aversive state and defensive behaviour to relieve this state (Proulx & Heine, 2010). Defensive responses during the pandemic may stem from the meaning violations it has induced (Proulx et al., 2012).

##### Existential Isolation and Loneliness

Social distancing as a containment strategy affects relationships and thwarts the need to belong in different ways. In addition, the concept of existential isolation relates to an irreconcilable divide that always remains between people because subjective experience can never be fully shared (Pinel et al., 2017; Yalom, 1980). Like other existential threats, not feeling related to others has been associated with defensive threat reactions like ethnocentric behaviour (Greitemeyer, 2012). Relationships represent an essential remedy against both existential isolation and loneliness. Because every human being is existentially alone, sharing that “aloneness” in loving relationships can alleviate the associated pain. While some evidence finds loneliness to have risen throughout the pandemic (Banerjee & Rai, 2020), other research finds that loneliness has affected such people that have already been lonely prior to the pandemic (Bu et al., 2020). Through widespread social distancing, the pandemic has likely contributed to objective social isolation, feelings of loneliness (Killgore et al., 2020), and, presumably, feelings of existential isolation.

#### Covid-19 Activates Existential Threats

**Figure 1:**
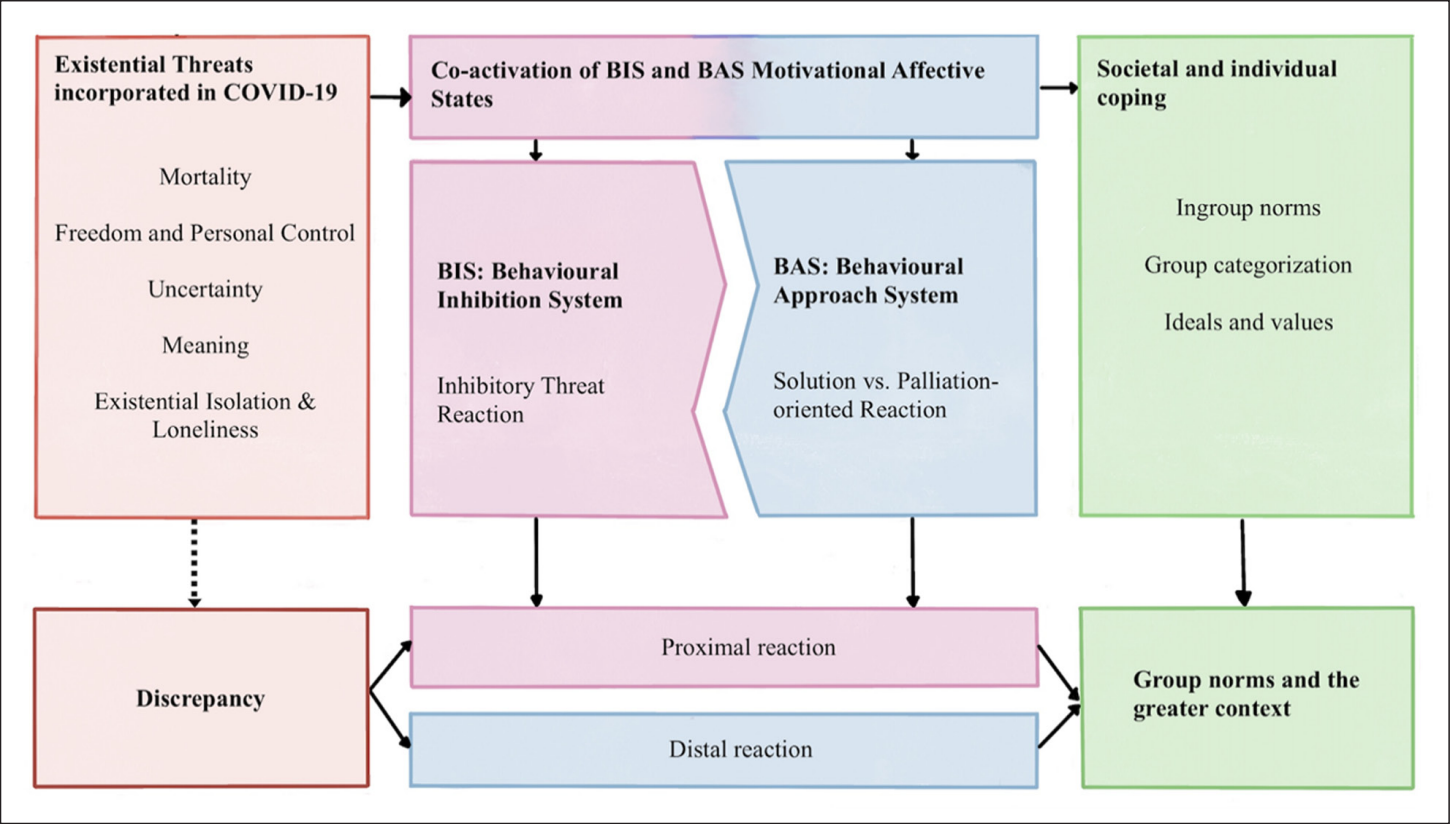
Application of the General Process Model of Threat and Defense to Covid-19 as a global threat. The Covid-19 pandemic activates existential threats (mortality, personal control, uncertainty, meaning, loneliness), such threats, in turn, are associated with proximal and distal reactions that ultimately form societal reactions to the threats.

The threat theories and dimensions described above refer to existential needs affected by the pandemic. Being confronted with many unmet needs likely leads to the perception of threat. We consider the GPMTD suitable to analyse the psychological consequences of the pandemic because it highlights the process behind threatened needs and people’s reactions to such threats. Moreover, it sheds light on the underlying process by providing an affective-motivational perspective on the dynamic between threat and defense (for an overview see Figure 1). Additionally, new data shows that different threat dimensions were activated simultaneously during the pandemic, with the unpredictability of the threat being the most prevalent (Klackl, 2023a). In addition, Covid-19 threat questions our moral foundations and cultural values, which are classic remedies against threat (Greenberg et al., 2003). Similarly, the pandemic also questioned our values and symbols by altering the way we lived – for example, prioritising safety over social norms and values as well as state forms and organisations. When moral foundations and cultural values are questioned or become unavailable, threat reactions are inevitable.

According to the GPMTD, motivational dynamics drive people’s attempts to overcome inhibition by engaging in distal defenses. Therefore, it is unique in integrating different streams of threat and defense literature, such as Terror Management Theory (Greenberg et al., 1997), the Meaning Maintenance Model (Proulx & Inzlicht, 2012), Uncertainty (Hogg, 2007), and Group-Based Control (Fritsche et al., 2013), to pinpoint a typical mediating process that explains a shared threat-and-defense cascade.^3^

### The Main Tenets of the GPMTD

From a psychological perspective, the pandemic is a global crisis that increases the salience of multiple existential threats. All these threats have in common that they heighten anxiety by activating the BIS. This generates an unpleasant state of aversive arousal that people strive to overcome by turning to various defenses.

#### Proximal Reactions to Threat

To better understand the affective-motivational state evoked by threat, we review evidence of BIS processes with various measurement techniques and BAS-related processes that appear as resolution or palliation efforts.

##### Neural Underpinnings of BIS

According to the GPMTD, existential threats activate brain regions associated with the BIS^4^ thereby increasing feelings of anxiety, physiological arousal, and vigilance processes (Jonas et al., 2014). The anterior cingulate cortex (ACC) is one of the key BIS regions in the human brain (Amodio et al., 2008; Gray & McNaughton, 2000). The ACC is sensitive to errors, conflicts, and surprises (Proulx & Inzlicht, 2012). Notably, the state of BIS is to be differentiated from the state of FFFS, which is activated in response to immediate and often physical *fear-inducing threats*. On the other hand, BIS is reactive to more psychological, imagined *anxiety-inducing threats* (Gray & McNaughton, 2000). The systems rely on different brain regions: Unlike fear and BIS activation, FFFS activation involves increased involvement of different brain regions (Quirin et al., 2012).

##### Physiological Arousal

Because of the causal link between the BIS and arousal that the GPMTD proposes, the peripheral nervous system provides another interesting avenue for studying threat and its downstream effects. Comparing an existential with a non-existential threat (Klackl & Jonas, 2019; Poppelaars et al., 2020) in several studies in a large array of cardiovascular, respiratory, and electrodermal parameters indicative of arousal, we concluded that existential and non-existential threats lead to comparable and transient increases in physiological activation notably increases in heart rate and respiratory rate, translated into a subsequent 6-minute period of reflection (Klackl & Jonas, 2019), indicating that a small amount of arousal caused by existential contemplation lingers on for several minutes. Using a different experimental design, Poppelaars et al. (2020) could not observe these increases in heart rate. However, affective changes were apparent after the confrontation with a threat. Together, our physiological evidence led us to conclude that actively responding to questions about threatening aspects of life induces affective changes in self-report measures. However, for the physiological changes after the confrontation, evidence is more inconclusive.

##### Subjective Threat-Related Arousal

Several studies indicate that subjective BIS-arousal is heightened as a response to pandemic threat in self-report measures (Jutzi et al., 2020; Reiss et al., 2020). We relied on bodily sensation maps to investigate whether people respond to threat with subjective arousal (Reiss et al., 2021). Participants were confronted with seven threat scenarios (mortality salience, uncontrollability, uncertainty, freedom restriction, identity, meaning threat, and social-evaluative threat). Bodily sensation maps following different threat saliences and a neutral control scenario showed reported sensations in the head, neck, and chest regions (Reiss et al., 2021). Results show that physiological and self-rated arousal diverge: Participants felt impaired subjectively. However, while we could observe neural changes in response to contemplating existential threats, we could not detect differences in the physiological parameters.

##### Proximal Activation of BAS

The GPMTD suggests that approach-oriented defenses occur distally. However, there is also evidence that proximal defense can be approach-related, as in the case of reactance (Mühlberger et al., 2020). Participants read restricting scenarios inducing self-experienced or vicariously experienced reactance (e.g., reactance experienced by a friend) or a neutral control condition. The results indicate that self- and vicariously experienced restricting scenarios elicited approach-related affect. Similar immediate approach reactions manifested during the pandemic with protesting in the streets against the restrictive measures imposed and breaking the lockdown rules to re-establish personal freedoms and, thereby, agency. This supports that deciding for oneself is directly rewarding, as demonstrated in EEG research where perceived control (arguably the flipside of uncontrollability) increased reward positivity (Mühlberger et al., 2017). In our structural equation model (see description below; Reiss et al., 2020), next to BIS and BAS, the motivational state of reactance was also strongly associated with social media use, a presumably approach-related behaviour.

#### Distal Threat Reactions

To escape from the uneasy state of BIS arousal, distal defense strategies are co-activated as a threat episode goes by, and threat salience vanishes (Pyszczynski et al., 1999). Defenses may vary from engaging in personal projects (Reiss et al., 2020) or ethnocentric tendencies (Agroskin et al., 2016) to conspiratorial ideation (Jutzi et al., 2020). Visible during the first wave of the pandemic in 2020, people were emptying supermarkets, supporting anti-Asian ideology (Lee, 2020) or falling prey to conspiracy theories (van Mulukom et al., 2020). As mentioned, distal responses can be categorised as those offering direct resolution (finding solutions to the problem) or palliation (dealing with the aversiveness of threat in an unrelated domain). Direct resolution may create long-lasting solutions (Park & Millora, 2012), whereas palliative defenses help to redirect approach motivation through the satisfaction of threat-unrelated needs. This includes ingroup support and outgroup derogation that are thematically unrelated to the threat (Jonas & Mühlberger, 2017). For a long time, the pandemic precluded a resolution response as neither vaccines nor cures have been widely available. As a consequence, we could and still can observe a trend towards palliative strategies (Reiss et al., 2020). Still, even when vaccines became available, several groups of people responded with resistance rather than getting the shot. We argue that this is most likely the case because these people did not perceive the vaccination as a resolution in the first place. Instead, the vaccine may have been perceived as a threat (with the fear of potential adverse long-term effects) or as not-aligning with people’s worldviews. For many people resistance against the vaccine was and is motivated ideologically (Bilewicz & Soral, 2022).

##### Clinging to One’s Ingroup as a Distal Defense

After encountering a threat, people are typically more prone to distinguish between their in- and outgroups (Fritsche et al., 2011). They tend to identify more strongly with their ingroup (Fritsche, 2022; Hogg, 2009), which serves the restoration of basic human needs on the social level of the self. These findings can be explained by theoretical approaches based on the social identity approach (Tajfel & Turner, 2004, Turner et al., 1987). According to self-uncertainty theory (Hogg, 2000, 2007), people regain a sense of self-certainty (that is, who they are and what they stand for) through self-categorising as group members when they feel uncertain as individuals. Group-based control theory (Fritsche et al., 2008; Fritsche, 2022) postulates a similar mechanism for the need for control: People, who experience a lack of control on the personal level of the self, can restore feelings of control on the social level of the self by thinking and acting as a group member (Fritsche et al., 2013). Acting as a group member can be expressed by complying with ingroup norms (Stollberg et al., 2017). People who experienced a personal lack of control increased their support for organisational change, innovative projects, and anti-right-wing protest when these were normative (approved of and followed by the majority) for their ingroup but not when they were normative for an outgroup (Stollberg et al., 2017). Support in line with ingroup norms following a threat to one’s personal sense of control can be interpreted as a demonstration of collective agency. Findings that show that identification with agentic ingroups following personal control threat increased perceptions of collective efficacy (Stollberg et al., 2015) support this notion.

In the face of a global crisis, such as the pandemic, different types of ingroups can become important to people to satisfy their needs and to deal with the crisis threat. These groups can range from close ingroups such as family and friends to larger self-categorisations such as humanity. Evidence from the pandemic suggests that one powerful ingroup that people identify with and act as part of is their national ingroup (Van Bavel & Boggio, 2020).

In sum, identifying with ingroups seemed to be particularly effective in coping with threats, as people can turn to intergroup distinctions when the personal self is threatened at any given time. Through identification with an ingroup, individuals can experience a sense of control, certainty or meaning and achieve a sense of stability and predictability in uncertain and threatening situations. Therefore, identifying and acting as a group member becomes an essential tool for managing crises.

##### Distal Defenses Observed During the Pandemic

**Figure 2:**
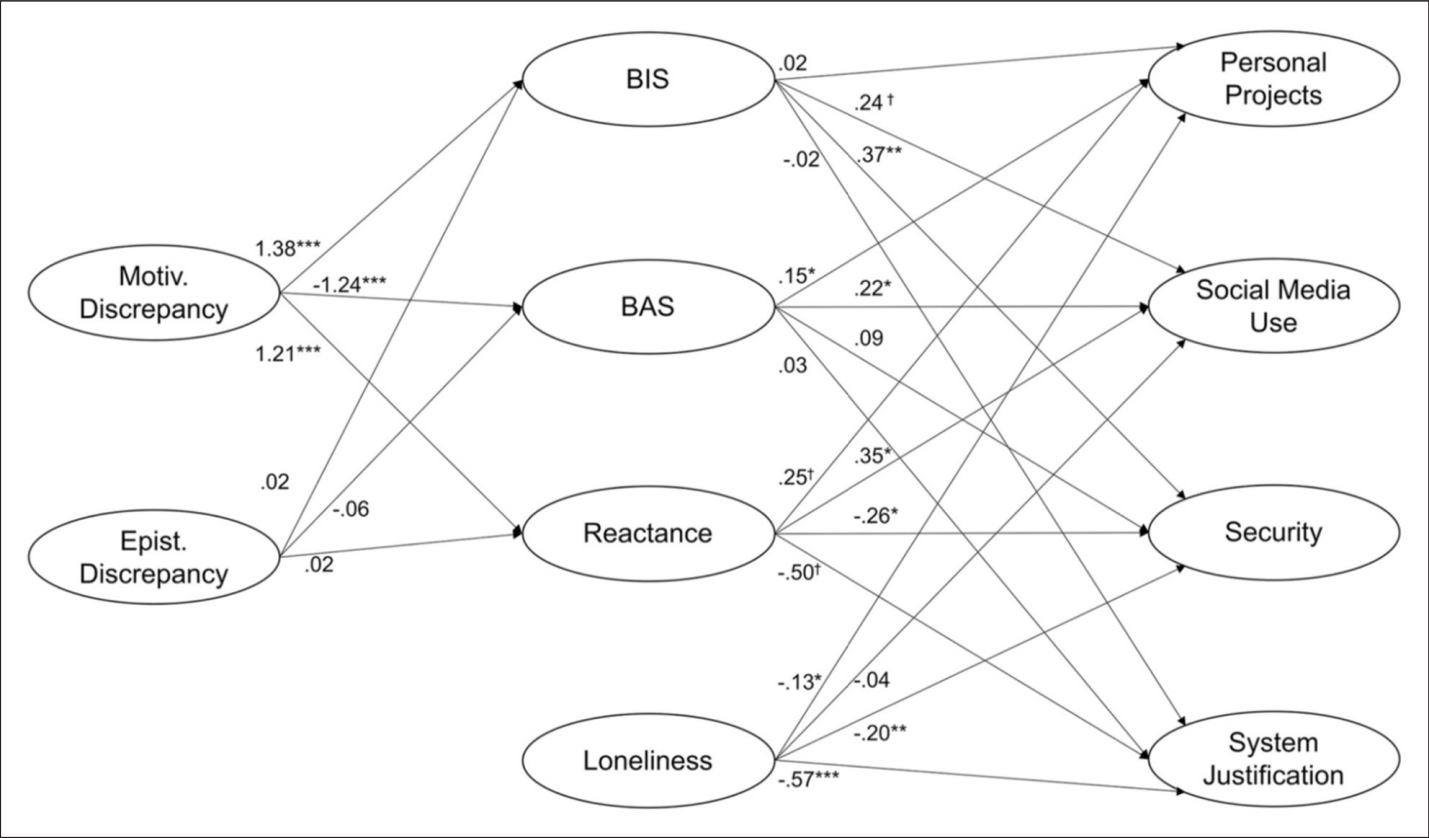
Motivational discrepancies elicited in the pandemic are associated with subsequent BIS, BAS and reactance affect. This affect again is associated with defensive reactions. From “From anxiety to action – Experience of threat, emotional states, reactance, and action preferences in the early days of COVID-19 self-isolation in Germany and Austria,” by S. Reiss, V. Franchina, C. A. Jutzi, R. Willardt and E. Jonas, 2020, *PLOS ONE*, 15(12), p. 1 (https://doi.org/10.1371/journal.pone.0243193). CC BY 4.0.

Recent studies have focused on these ingroup effects as palliative defense strategies in the pandemic. Reiss et al. (2020) investigated a possible link between peoples’ Covid-19 threat perception and their use of four different defensive strategies. They conducted a survey (*N* = 395) within the first days of implementing social distancing measures in Austria and Germany during the first wave (March 2020). Participants first indicated how threatened they felt on a Covid-19 threat perception scale (sample item: *“The Coronavirus determines most of what I can and cannot do”*). Participants then indicated their affective states. Structural equation modelling (see Willardt et al., in press, for structural equation modelling and the GPMTD) revealed a significant positive path between motivational discrepancy and BIS-related affect (i.e., anxiety). Furthermore, the association between motivational discrepancy and security-related strategies (such as the willingness to wash hands frequently) was mediated via increased BIS affect. Interestingly, reactance (predicted by discrepancies) as a proximal motivational-affective state was positively associated with social media use and personal projects but negatively associated with system justification. Thus, motivational discrepancies are core to understanding the pandemic threat dynamic (see Figure 2 for the whole model).

Focussing on abstract defenses, Jutzi et al. (2020) measured Americans’ perceptions of Covid-19 as a motivational discrepancy as well as their BIS activation. Following participants indicated whether they would vote for a Republican or a Democratic candidate, how much they felt their chosen party to be a strong unity and how motivated they were to support that political party as a distal defense. Results showed that the effects of perceived motivational discrepancies on distal defenses were mediated by increased BIS activation; in other words, threat increased anxious affect which in turn predicted defensive reactions.

To corroborate the findings, we manipulated Covid-19 threat salience (*N* = 348) where participants read a set of statements that emphasised the fact that there is no cure against Covid-19 (*threat –* experimental) or read a set of unrelated facts that had the same graphic materials (control). The results indicated that higher perceived threat (measured as a manipulation check after the manipulation) was related to increased *ingroup entitativity, system justification*, and *conspiratorial beliefs (about an outgroup)*, all again mediated by increased BIS activation. In our analysis we used the manipulation check as a mediator, the effect is not apparent when using the manipulation. These results show that proximal BIS activation foreshadows distal defenses (Jutzi et al., 2020).

#### Critical Reflection on the Model

##### Questioning the Unified Perspective

Even though most studies support the unified perspective of the GPMTD, some evidence from our lab shows that different threats have unique properties that influence the details of threat processing. Reiss et al. (2021) tried to distinguish between different psychological threats by exposing participants to seven different threat saliences (mortality, social evaluative, freedom, identity, uncertainty, uncontrollability, and meaninglessness) and a neutral TV salience. The results indicate that all threat saliences differed from the neutral TV manipulation in various descriptive and affective dimensions. The individual profiles of the various threats allowed a classifying algorithm to identify the type of threat manipulation with above-chance accuracy. Further analysis showed three threat clusters: Mortality as one cluster, freedom and meaning threat as a second, and uncontrollability, uncertainty, and isolation as a third threat cluster. Hence, in addition to the general threat and defense undertones, different threats seem to have distinctive overtones. While machine learning algorithms can tell what existential topics are on people’s minds based on their written outputs, individuals seem to be bad at telling what existential concerns they are concerned with at a given time. More recent evidence indicates that when people are threatened by one existential threat, they often are preoccupied with other existential threats as well (e.g., feeling lonely and uncertain simultaneously; Klackl et al., 2023a). This may be due to the general inability of individuals to differentiate and report an unspecific sense of threat in self-disclosure. Focusing on the differences in addition to the commonalities of different threats will be an integral part of our future projects.

##### Sequence Effects

The original version of the GPMTD (Jonas et al., 2014) assumes that experiencing a discrepancy initially leads to BIS activation, followed by subsequent BAS activation. Recent evidence supports this assumption, while threat is associated with proximal BIS activation, engaging in a defense distally promotes BAS activation in comparison to a control condition (Stollberg, 2023). Next to this distal BAS activation recent findings indicate that individual affective approach reactions may immediately follow a discrepancy. People often report proximal approach-related affect in the form of anger after personal freedom restrictions. At the same time, participants who enter the defense process with a high BAS state (i.e., a positively activated state) appear to make the use or acceptance of defensive behaviour more likely (Klackl et al., 2023b). Future studies should investigate order effects within the threat and defense cascade in more detail.

##### Potential General Buffers

Other research from our group indicates that for specific populations distinct defensive strategies may be more important than others: First-generation university students (FGS), that are arguably disadvantaged, showed less trust in the overall university system than continuous-generation students. Interestingly, FGS relied more on personal contact to cope with the threat of Covid-19 (Möller et al., 2021). This more general resource, which one may call an anxiety buffer, was not available within pandemic circumstances due to contact restrictions. Thus, FGS were even more disadvantaged. Analysing and finding general resources and anxiety buffers specific to different populations is thus an important task for future studies.

### Opportunities Resulting from Pandemic Research

We have learned that the Covid-19 pandemic activated existential threats in many aspects of people’s lives. This perception influenced and influences cognitive (vigilance), affective (anxiety), and motivational (inhibition of goal pursuit) processes. The pandemic created and creates anxiety that requires management, either by resolving the anxiety-eliciting problems or by reactivating approach motivation. While threat resolution facilitates ongoing goal pursuit, palliation also provides a motivational gain by restoring feelings of positive determination. Based on the steps in the GPMTD, one might create interventions that target the threat by mitigating threat perceptions, satisfying underlying needs, and continuing to make new defenses salient and attractive. We now discuss how the pandemic can be dealt with in a way that optimises outcomes for individuals and societies (see Figure 1 for an overview).

#### Threat Perception

##### Threat Perception (Discrepancy Detection)

The first stage in the GPMTD describes the detection of a discrepancy. There was no solution to pandemic threat for a long time. Yet, how information around pandemic restrictions and measures is communicated, influenced, and influences the overall appraisal of the pandemic situation. Guidelines on how to communicate Covid-19-related threat information according to personal needs thus advise a “delicate combination of providing rules and structure in a caring and autonomy-supportive way” (Martela et al., 2021, p. 1). By adopting such communicative approaches, authorities can increase citizens’ feelings of cognitive control by explaining why certain decisions are being made. Moreover, transparency in communication, and accountability, have positive effects on both quality of decision-making and information-seeking processes (Jonas et al., 2008).

#### Threat and Defense Cascade

##### Addressing BIS-Activation and Related Anxiety by Satisfying Underlying Needs

Since the pandemic was and is complex and overwhelming, proximal affective threat reactions, such as BIS-activation accompanied by anxiety, *evolve and must be addressed*. BIS-induced anxiety can best be dealt with by addressing its underlying needs. Taking from the prior analysis of Covid-19 as an existential threat, we learned that underlying basic, fundamental needs such as certainty, control or meaning are associated with BIS-related anxiety. Thus, satisfying basic needs is a key ingredient in counteracting the vicious cycle of threat on dispatched information selection that can lead to “exclusionary [ethnocentric] threat-responses” (Lueders et al., 2016, p. 1477). Participants given an imaginary opportunity to voice their needs displayed significantly less negative affect and less avoidance motivation in change situations (Reiss et al., 2019). It lies within the nature of existential threats, that the matching underlying need cannot be satisfied. One strategy to cope with these circumstances is to identify, even within the limited circumstances of the pandemic, other needs that can (at least partially) be satisfied. Giving people the opportunity to satisfy such unrelated needs (as a palliative strategy; for example, restructuring the daily routine), may be one strategy to cope with the overall pandemic situation better.

##### Accepting Anxiety

As just mentioned, most anxiety elicited by a global threat could and cannot be addressed directly. Thus, strategies to accept anxiety may need to be implemented (Weis et al., 2021; Simonsson et al., 2021). To address a threat, one option is to encourage conscious acceptance of it. However, to temper expectations, clinical research shows that the effect of acceptance interventions on anxiety is small to moderate (Aldao et al., 2010). Additionally, other clinical practices such as mindfulness should be considered to cope with anxiety stemming from the pandemic (Vøllestad et al., 2011).

This can be accompanied by self-compassion (Neff et al., 2003) building on three main premises: practising self-kindness (instead of self-judgement and being one’s enemy); understanding that one is not alone in experiencing anxiety (and sharing the experience with others); and consciously understanding what is happening and why (instead of over-identifying with the threat).

#### Palliative Defenses to Manage Threat

Even if people can reduce anxiety to some extent, palliative responses are often needed to help regain approach motivation. Some palliative strategies are detrimental to society even if they provide anxiety reduction for the individuals using them: Because people’s worldviews and values are varied and often incompatible, palliation based on defending values may aggravate societal tensions between groups and amplify societal division and discord (Reiss & Jonas, 2018). Providing individuals and societies with useful palliative strategies is thus vital to fight the pandemic.

##### Choice of Ingroup Membership

Membership in social groups can be a resource in the face of threat: When confronted with an existential threat, people rely on social groups, show increased identification with self-defining ingroups, and demonstrate conformity to salient ingroup norms (e.g., Fritsche et al. 2013; Hogg, 2007; Jonas et al., 2008; Stollberg et al., 2017). To constructively manage a crisis such as a pandemic within a society, naturally, it becomes essential which specific ingroup individuals identify with and of which beliefs and behaviour they approve. The attractiveness of social groups generally can depend on the threatened need. For example, extremist groups are preferred for the satisfaction of self-certainty (Hogg et al., 2014), and agentic groups are preferred to affirm control perceptions (Stollberg et al., 2015). We already know that when people identify with an ingroup, this identification is associated with higher approval of ingroup norms, and thus the availability of a specific ingroup is vital. This even extends to change norms: when innovations and changed norms are approved by relevant ingroup members (Stollberg et al., 2017), change norms may promote innovative behaviours. A prominent example in the pandemic was prosocial ingroup norms celebrating the value of medical personnel. Hence, emphasizing the significance of relevant ingroup memberships to facilitate effective pandemic management was and is essential to effective pandemic management.

Additionally, fostering the positive communication of change norms and creating external incentive structures for innovation should help shift the focus towards the future in crises such as the pandemic.

##### Dealing with Different Social Identities

As ingroup norms, goals, and ideals shape behaviour in the face of threat, which groups people identify with becomes vital. Disentangling different group categorisation effects during the pandemic is complex and supranational alliances such as the EU depend on whether national or supranational identities are activated. Identification with a nation predicted people’s endorsement of and adherence to national public health measures (Van Bavel & Boggio, 2020), although, traditionally, nationalism has been associated with problematic outcomes such as hostility towards the outgroup (Druckman, 1994). This demonstrates that national identification may be helpful in managing crises such as the current pandemic and climate change, which have national and global ramifications. A cautionary factor is that, while national identification can bring benefits, identification with an ideally inclusive large ingroup is highly advisable to prevent the frequent and undesirable devaluation of outgroups. Identity complexity theory suggests that when people have a more complex, more flexible identity structure, they are less threatened by stereotype threat; that “[s]ocial identity complexity reflects the degree of overlap perceived to exist between groups of which a person is simultaneously a member” (Roccas & Brewer, 2002, p. 88). This identity might integrate a European identity and a national identity to confront Covid-19.

There may be positive effects of global threats alongside these divisive forces. In the case of the complex global threat of Covid-19, studies from our group have shown that manipulating the Covid-19 threat increased both ingroup and outgroup warmth and competence ratings (Jutzi et al., 2020). This suggests a unifying potential for society when identification with superordinate groups of all humans becomes attractive as a palliative defense (Reese et al., 2015).

## Outlook

### The Covid-19 Pandemic as an Exemplary Global Threat

Describing the pandemic as an exemplary global threat showed that different existential threats were activated during the pandemic, and the GPMTD may help to explain affective-motivational consequences arising from these threats. Recent evidence suggests that the processing of a threat follows an affective-motivational cascade starting with an inhibitory affective component that translates into approach-related affective reactions accompanied by defensive reactions and behaviours. This insight may help to understand future and current crises – be it migration, globalisation, or the climate crisis and pay attention to the affective reactions such crises elicit.

Looking at the climate crisis, we can find that both the pandemic and the climate crisis activate existential threats. At the same time, reactions to the climate crises resemble reactions to the pandemic: People react with palliative defenses such as ethnocentrism (Uhl et al., 2018) and outgroup derogation (Barth et al., 2017). Global crises, therefore, appear to activate psychological threats associated with certain defensive behaviours. Future research should examine the connection between affective reactions and defensive responses to global crises such as climate change threats, migration, or brutal conflicts.

This becomes particularly relevant since attempting to purely transfer coping strategies from the pandemic to the climate crisis cognitively, was not helpful in other research: In a recent study, Ecker et al. (2020) investigated the relationship between climate change and pandemic concerns. Here, framing the pandemic as a “trial run” to learn how to solve climate issues did not help enhance participants’ investment towards climate change issues.

We hypothesise that instead, individuals may require a longer learning journey to process the affective reaction triggered by existential threats, acquire strategies (for instance, such that we presented in the current article) to cope with these threats, and ultimately integrate the experience and coping strategies and transfer them to different crises. The affective threat reaction observed with Covid-19 may be generalisable to other global threats and thus give perspective into addressing them scientifically (Willardt et al., in press). More than that, we must learn at various stages of threat processing as a collective or group and address future crises with a new understanding. The GPMTD may provide one perspective for looking at such global threats. This can result in individuals and societies tackling the pandemic as an “unprecedented opportunity to rebuild and re-imagine” the future (Fore, 2021).

## Footnotes

Different theories use different vocabulary to describe the experience of threat: Dissonance theory describes conflicting cognitions (Festinger, 1957), while the Meaning Maintenance Model (Proulx & Inzlicht, 2012) describes threats as meaning disruptions, Self-discrepancy theory revolves around discrepancies in the self (Higgins, 1989). The definition of discrepancy may be the smallest common denominator to fit these concepts into one definition.

The distinction between “problem-focused coping (active coping, planning)” (Carver et al., 1989, p. 267) and “emotion-focused coping (seeking of emotional social support, positive reinterpretation)” (Carver et al., 1989, p. 267) was described earlier in time (Carver et al., 1989; Lazarus & Folkman, 1984). Within the GPMTD, these approach-oriented responses can be classified as threat-related *direct resolution* (*“problem-focused coping”*) versus threat-unrelated *palliation* (*“emotion-related coping”*). However, the concepts differ substantially: Emotion-focused coping entails remaining in and working on a threatening situation. Palliation, conversely, entails distracting oneself from the threatening situation and focusing on threat-unrelated matters.

The GPMTD differs from other theories by describing the process from threat to defense, integrating other existential threat theories. Terror Management Theory (TMT) distinguishes between proximal and distal defensive mechanisms (Pyszczynski et al., 1999). This proximal/distal distinction is also featured in the GPMTD. In the Meaning Maintenance Model (MMM, Proulx & Inzlicht, 2012), fluid compensation describes defense in a threat-unrelated domain. It thereby makes a distinction between palliative and solution-oriented responses to inconsistencies. This distinction is echoed in the GPMTD’s distinction between palliative and solution-oriented distal defense. Inspired by Reactive Approach Motivation (RAM) theory, GPMTD takes up the mediating function of BIS (McGregor et al., 2010) and describes a pathway from threat to distal threat responses. Finally, even though TMT incorporates motivational drivers into the process, the GPMTD embraces the motivational and affective nature.

The state of anxiety that the GPMTD attributes to the BIS has been given various names by different social psychological theories of threat and defense: The Meaning Maintenance Model (Proulx & Inzlicht, 2012, Heine et al., 2006) calls it *disanxiousuncertlibrium;* Terror Management Theory refers to it as *a potential for anxiety* (Greenberg et al., 2003, p. 516); Reactive approach motivation theory calls it *anxious uncertainty* (McGregor et al., 2010). The GPMTD attributes these states to the BIS, an *evolutionarily primitive anxiety system* (Tritt et al., 2012, p. 715).
